# Supplementary material for: CD4+T-cells create a stable mechanical environment for force-sensitive TCR:pMHC interactions
Source: Nat Commun. 2025 Aug 15;16:7577. doi: 10.1038/s41467-025-62104-2 (PMC12354883; doi:10.1038/s41467-025-62104-2)
Supplement: Supplementary file 1 — Supplementary Information [file 41467_2025_62104_MOESM1_ESM.pdf]

CD4+ T-cells create a stable mechanical environment for force-sensitive TCR:pMHC interactions

Supplementary information

Lukas Schrangl[1,2], Florian Kellner[3,9], René Platzer[3,10], Vanessa Mühlgrabner[3], Paul Hubinger[3], Josephine Wieland[4], Reinhard Obst[4], José L. Toca-Herrera[1], Johannes B. Huppa[3,6,7,8], Gerhard J. Schütz[2], Janett Göhring[3,5*]

[1] Department of Bionanosciences, Institute of Biophysics, University of Natural Resources and Life Sciences, Vienna, Austria
[2] Institute of Applied Physics, TU Wien, Vienna, Austria
[3] Institute for Hygiene and Applied Immunology, Center for Pathophysiology, Infectiology and Immunology, Medical University of Vienna, Vienna, Austria
[4] Institute for Immunology, Biomedical Center, Medical Faculty, Ludwig-Maximilians-Universität München, Planegg-Martinsried, Germany
[5] Department of Biotechnology and Food Science, Institute of Molecular Biotechnology, University of Natural Resources and Life Sciences, Vienna, Austria
[6] Institute of Cancer Immunology, Charité - Universitätsmedizin, Berlin, Germany
[7] Max Delbrück Center, Berlin, Germany
[8] German Cancer Consortium (DKTK), Heidelberg, Germany
[9] Valdospan, Tulln, Austria
[10] Institute for Pharmacology, Johannes Kepler University, Linz, Austria
[*] Corresponding author. Email: janett.goehring@meduniwien.ac.at

Contents

1 Supplementary figures 2

2 Supplementary tables 11

1 Supplementary figures

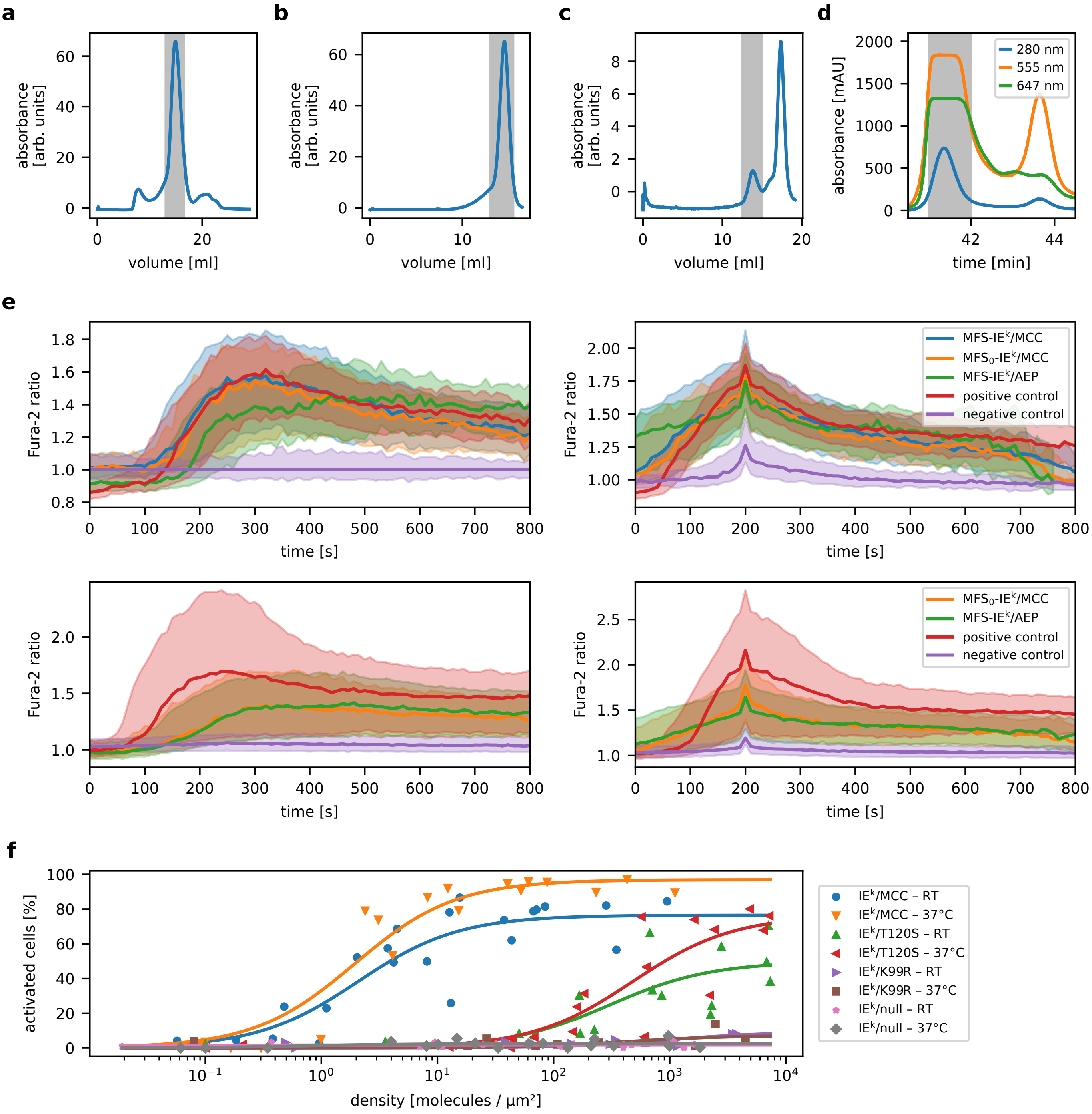

Supplementary figure 1: *Synthesis and functional validation of the AEP-IE^k molecular force sensor.* (a) Refolded murine pMHC class II molecule IE^k with AEP peptide (IE^k/AEP) was purified by Superdex 200 10/300 GL (S200) size exclusion chromatography. The grey areas correspond to collected fractions. (b) Post conjugation, the surplus of DBCO was removed from monomeric IE^k/AEP-DBCO via Superdex 75 10/300 GL (S75) gel filtration. (c) Unconjugated MFS was separated from MFS-IE^k/AEP via S75 gel filtration. (d) Absorbance profiles of HPLC purified Alexa 555/Alexa 647 dual-labeled AEP peptide confirmed successful label conjugation. (e) Calcium flux analysis of the SLBs decorated with MFS-IE^k/MCC, MFS-IE^k/AEP, and unlabeled MFS$_0$-IE^k/MCC, as well as ICAM-1 and B7-1. Positive control: ICAM-1, B7-1 and his-tagged IE^k/MCC. Negative control: only ICAM-1 and B7-1. Two independent experiments performed at room temperature are shown (upper and lower panel). The upper panel includes 504 to 1877 cells per condition. The lower panel includes 152 to 331 cells per condition. Shaded area represents the interquartile range of the population. (f) Measurement of T-cell sensitivity in room temperature vs. 37 °C using fluid-phase SLBs with his-tagged IE^k presenting the strong agonistic MCC, weak agonistic T102S, antagonistic K99R, and Null peptide. Calcium response was measured for varying antigen densities. For comparability, data sets for varying temperatures were measured on the same day and data was pooled from three independent experiments. Each condition includes the results from 430 to 3129 cells, with a total sum of 5706 cells (RT) and 3585 cells (37 °C) for IE^k/MCC, 4566 cells (RT) and 3776 cells (37 °C) for IE^k/T102S, 2469 cells (RT) and 1153 cells (37 °C) for IE^k/K99R, and 5519 cells (RT) and 1990 cells (37 °C) for IE^k/null. Source data are provided as a Source Data file.

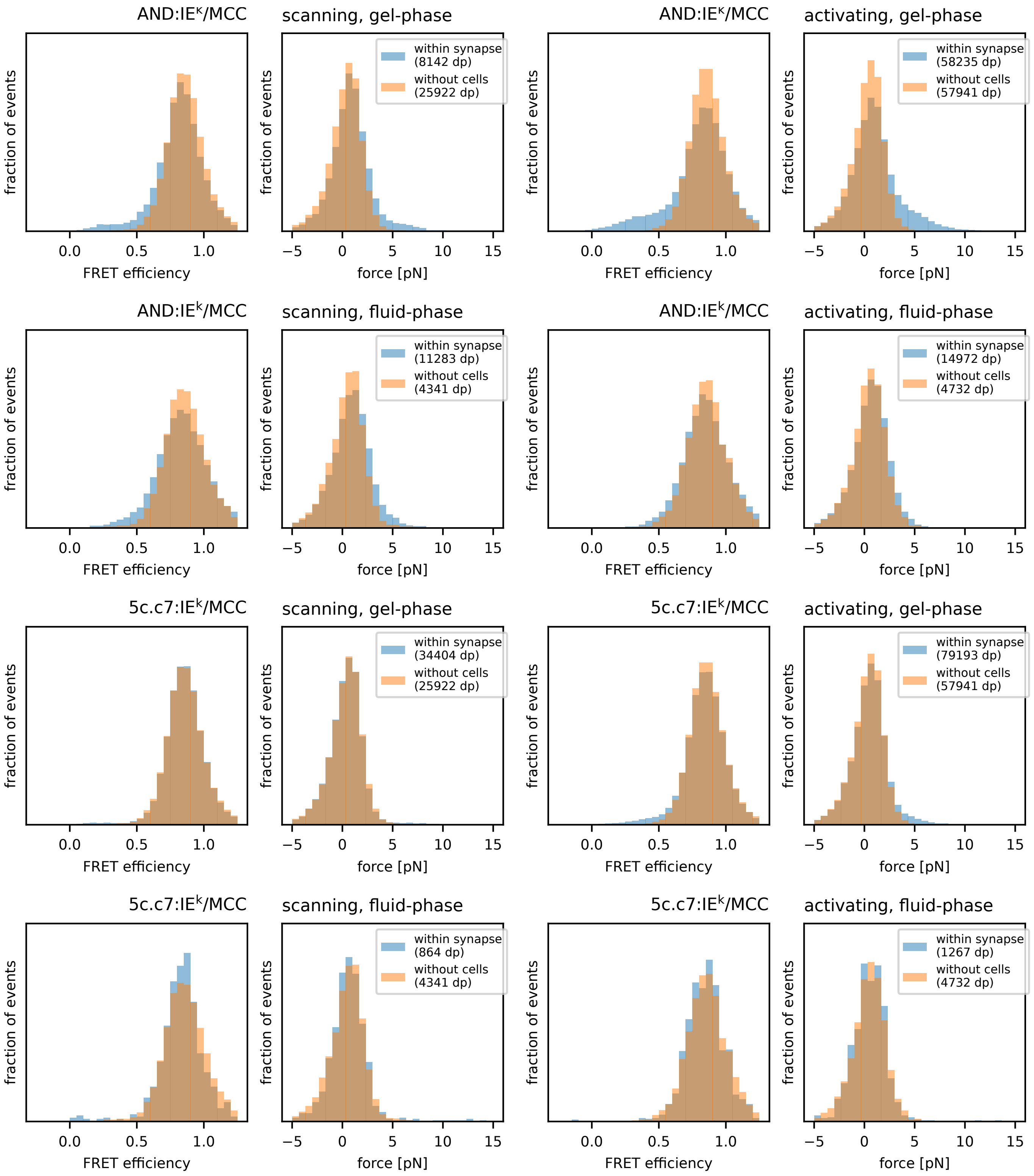

Supplementary figure 2: Continued on next page.

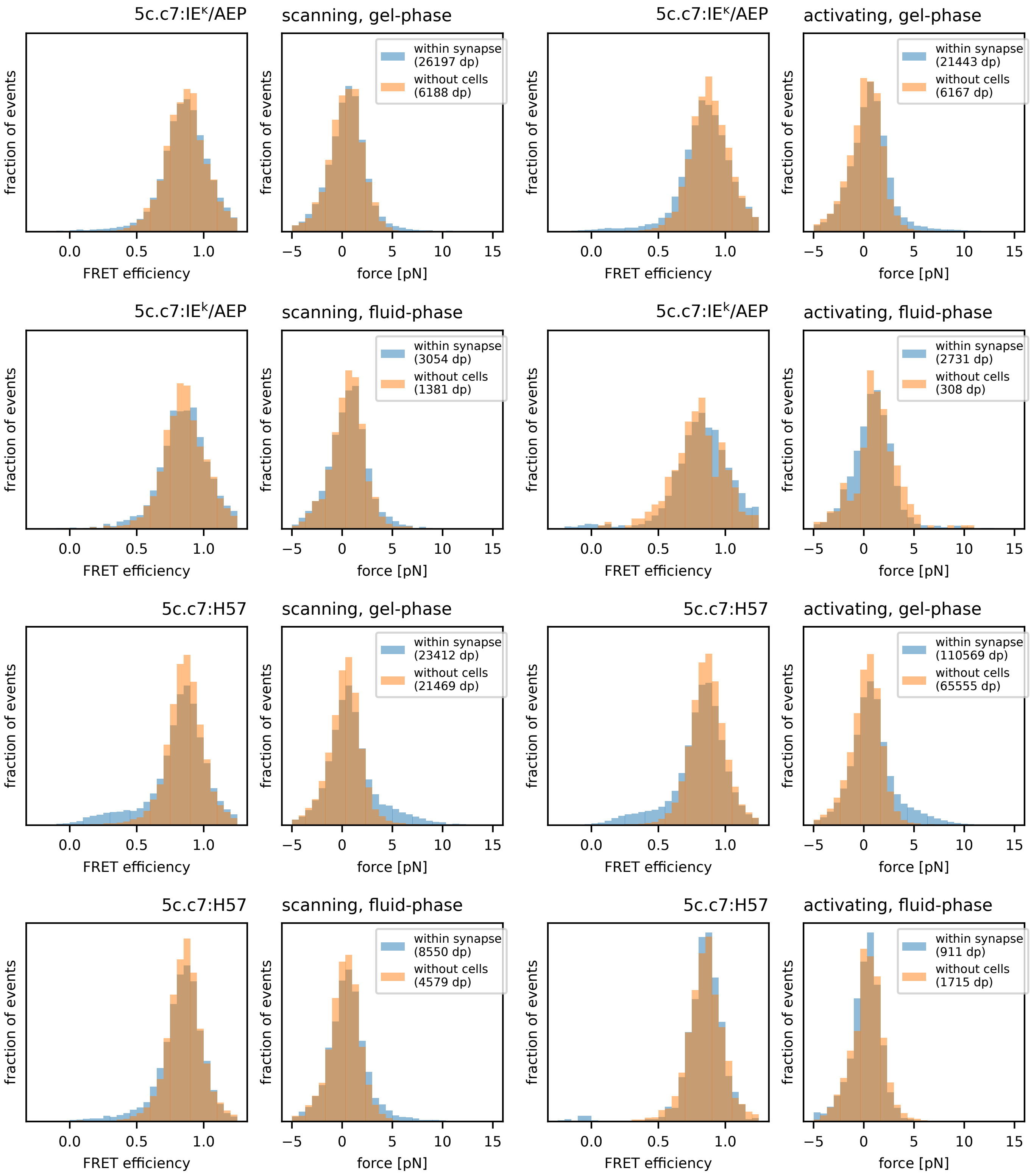

Supplementary figure 2: *FRET efficiency and force histograms of the single-molecule FRET trajectories* for scanning (left side of the panel) and activating conditions (right side of the panel) on gel-phase and fluid SLBs. The FRET efficiency histograms are plotted in the first and third column, whereas the respective force histograms are plotted in the second and fourth column. The number of data points participating in the analysis are mentioned in the legend, and the number of recorded trajectories and mice are summarized in supplementary table 1. Histograms are normalized such that the sum of bars equals one. Y axis scale is shared among all FRET efficiency histograms and among all force histograms. Source data are provided as a Source Data file.

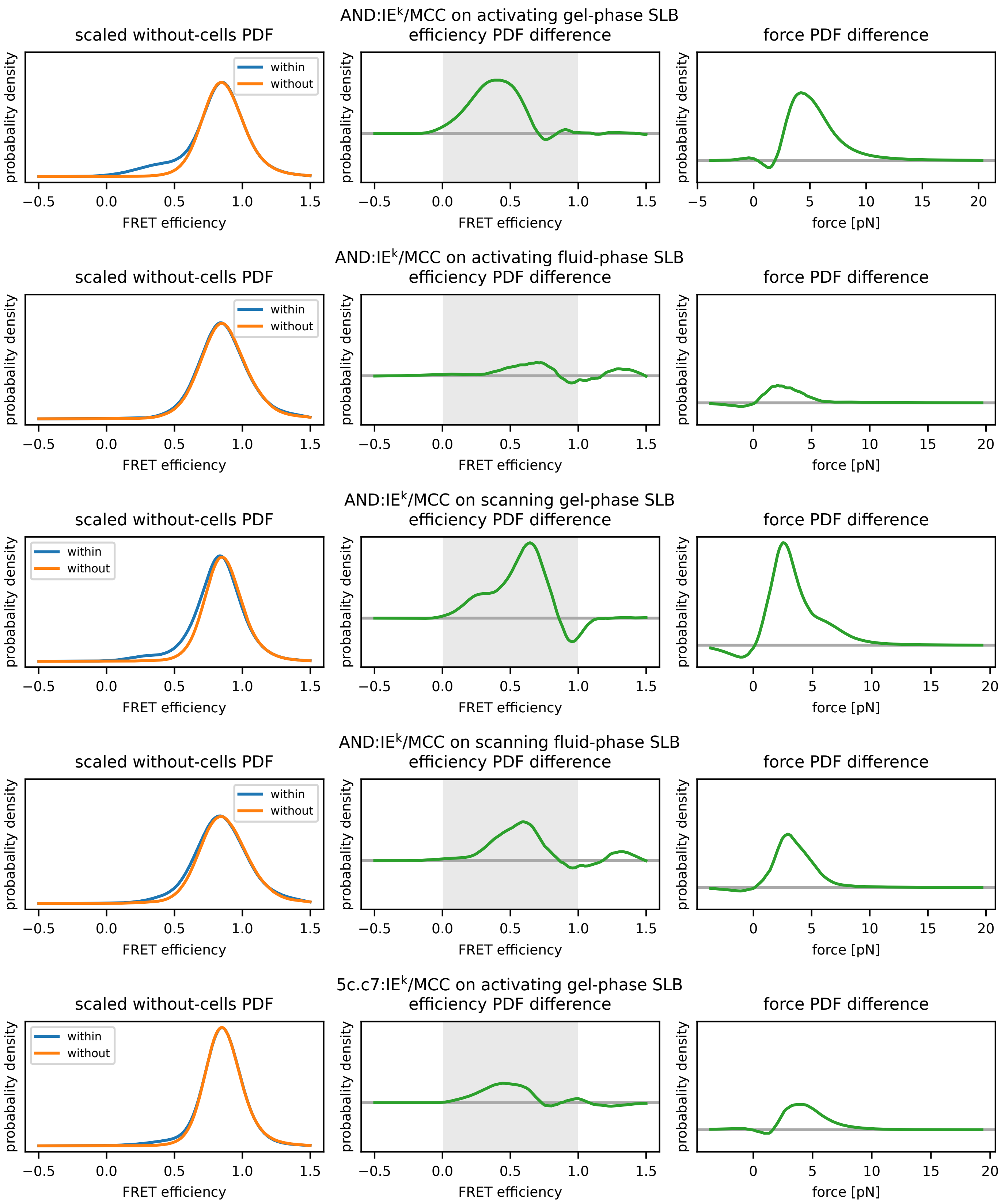

Supplementary figure 3: Continued on next page.

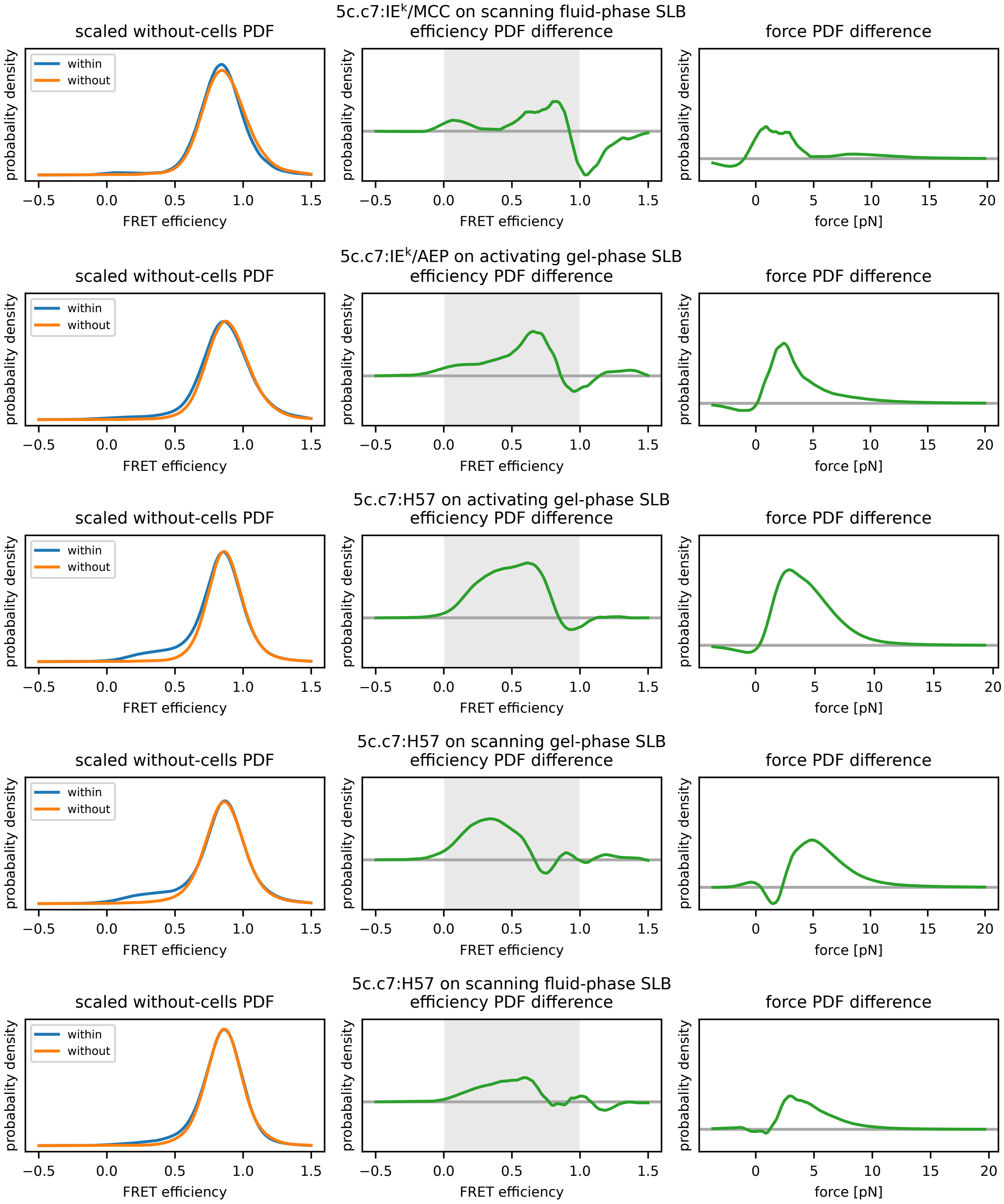

Supplementary figure 3: *Extraction of the low frequency force events from the entire population of FRET trajectories via PDF subtraction of the no-cell data.* Left panels: FRET efficiency histograms of raw data. Middle panels: Result of the PDF subtraction. Right panels: Conversion of the low FRET population into force. The number of recorded trajectories and mice are summarized in supplementary table 1. Y axis scale is shared among all FRET PDFs, among all efficiency PDF differences, and among all force PDF differences. Source data are provided as a Source Data file.

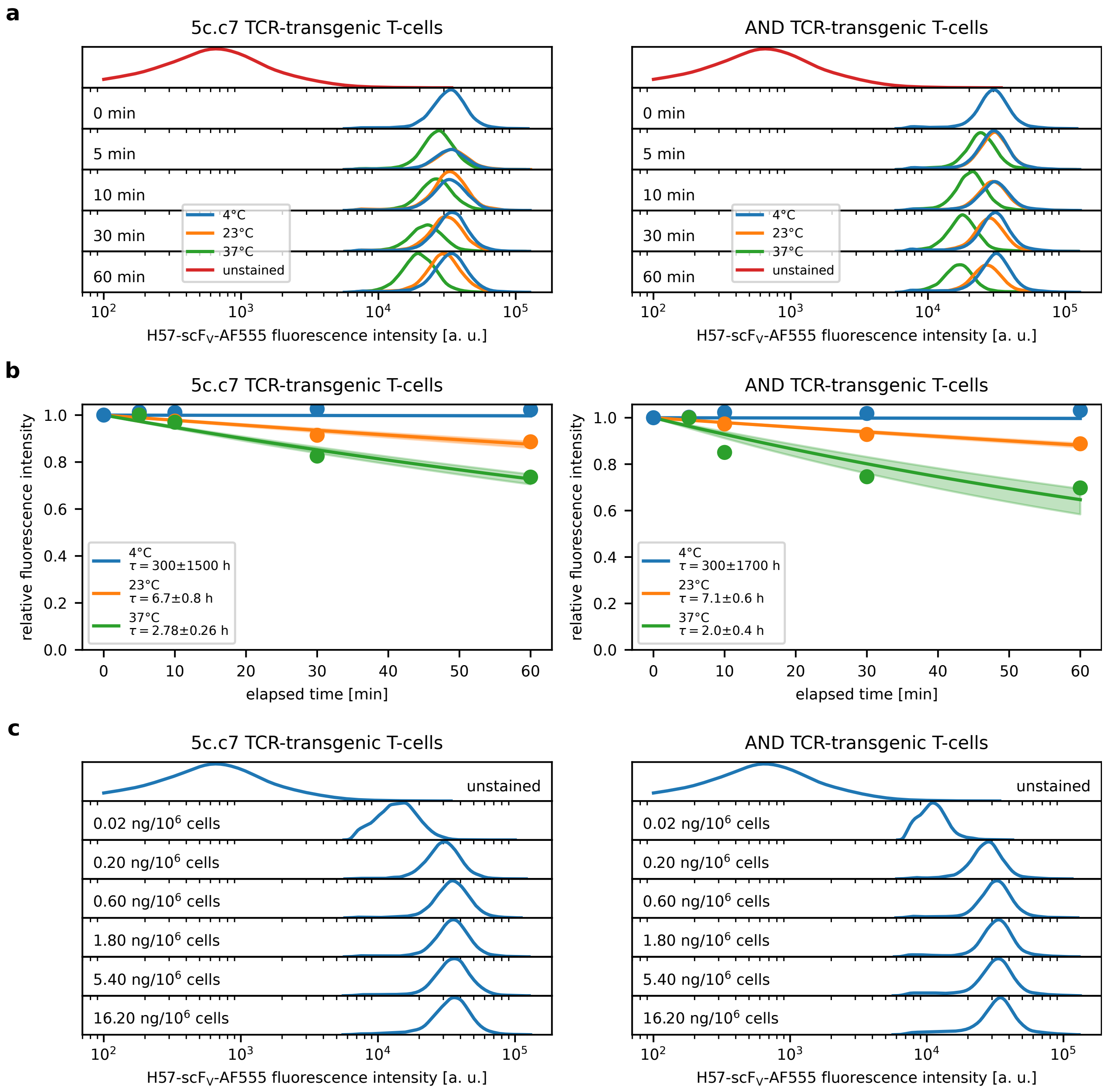

Supplementary figure 4: *Flow cytometry data for the label stability and saturation of transgenic T-cells with fluorescent H57-scF*$_V$. (a) 5c.c7 and AND T-cells were labeled and incubated for up to 1 hours in 4 °C, 24 °C and 37 °C. Label density was detected via flow cytometry. (b) Lifetime estimation of bound label for data in panel (a). Data points were fitted with a one phase decay curve with a plateau constraint of 0.1. Standard errors of the fit are depicted by the shaded areas. Each line represents approximately 50000 cells from one experiment. (c) Label titration to achieve saturation using 5c.c7 and AND T-cells. Source data are provided as a Source Data file.

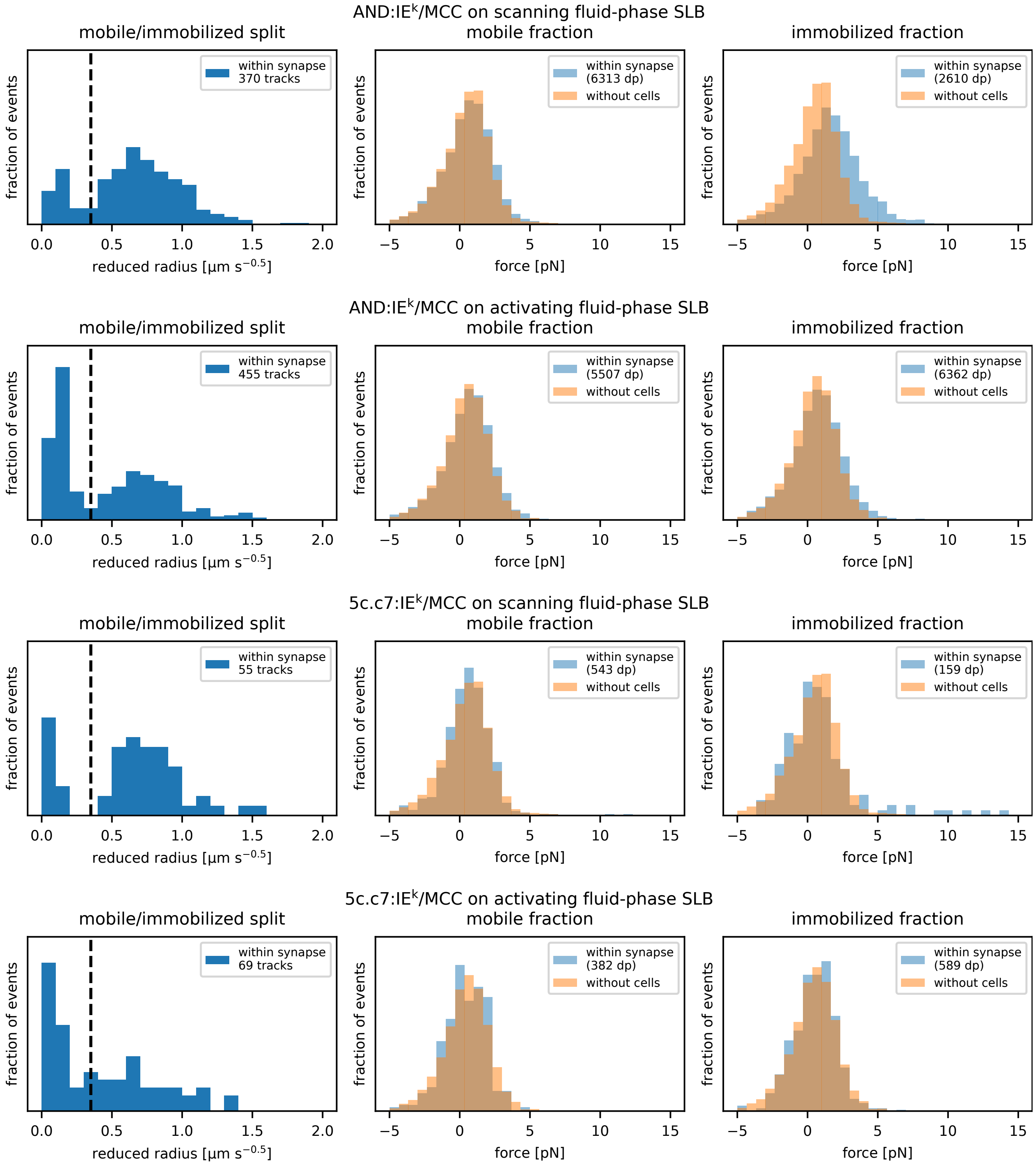

Supplementary figure 5: Continued on next page.

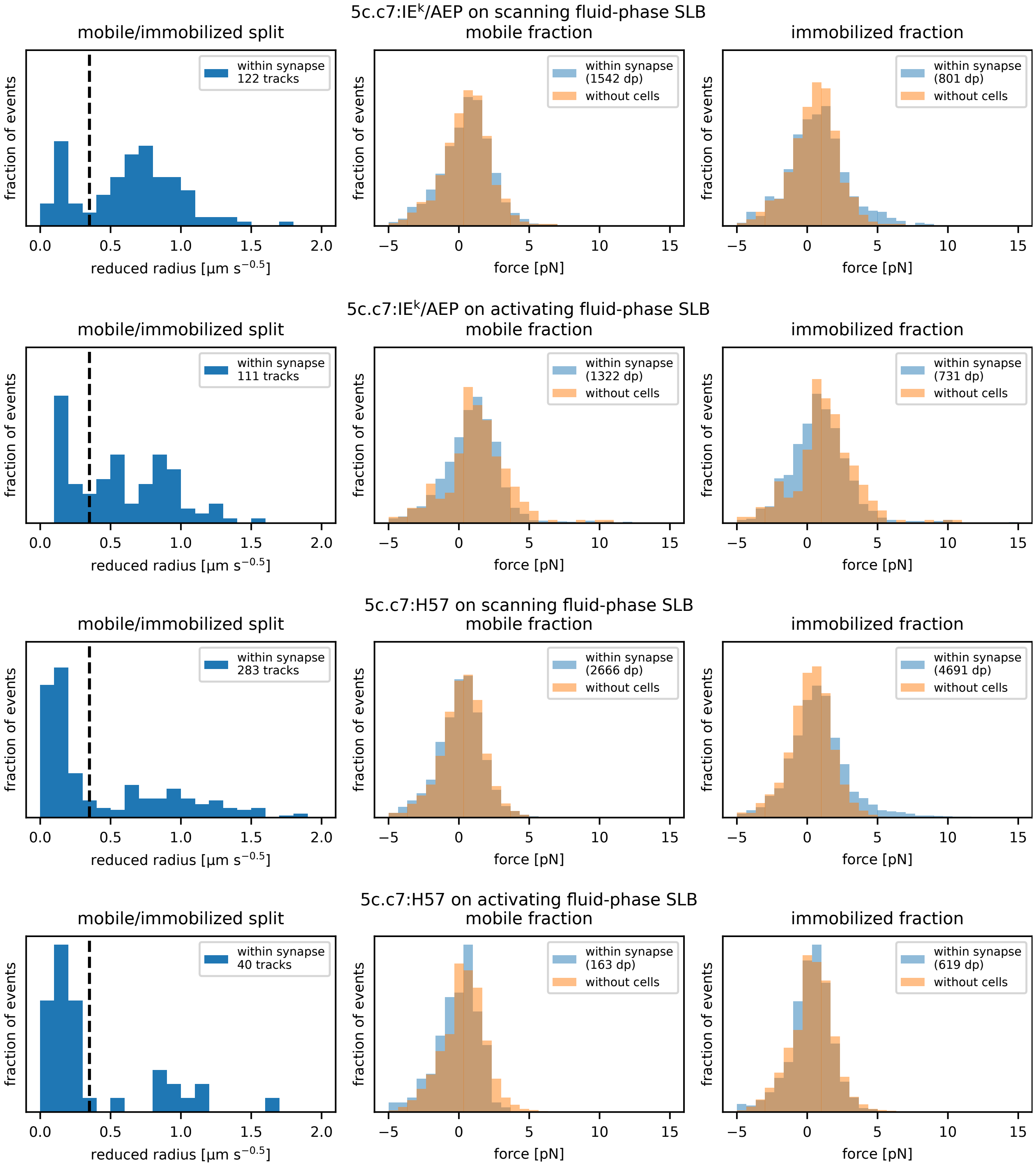

Supplementary figure 5: *Force histograms of the mobile and immobilized fraction and the respective immobilizations plots.* The number of trajectories and data points participating in the analysis are mentioned in the legend. Histograms are normalized such that the sum of bars equals one. Y axis scale is shared among all radius histograms, among all FRET efficiency histograms, and among all force histograms. The number of recorded trajectories and mice are summarized in supplementary table 3. Source data are provided as a Source Data file.

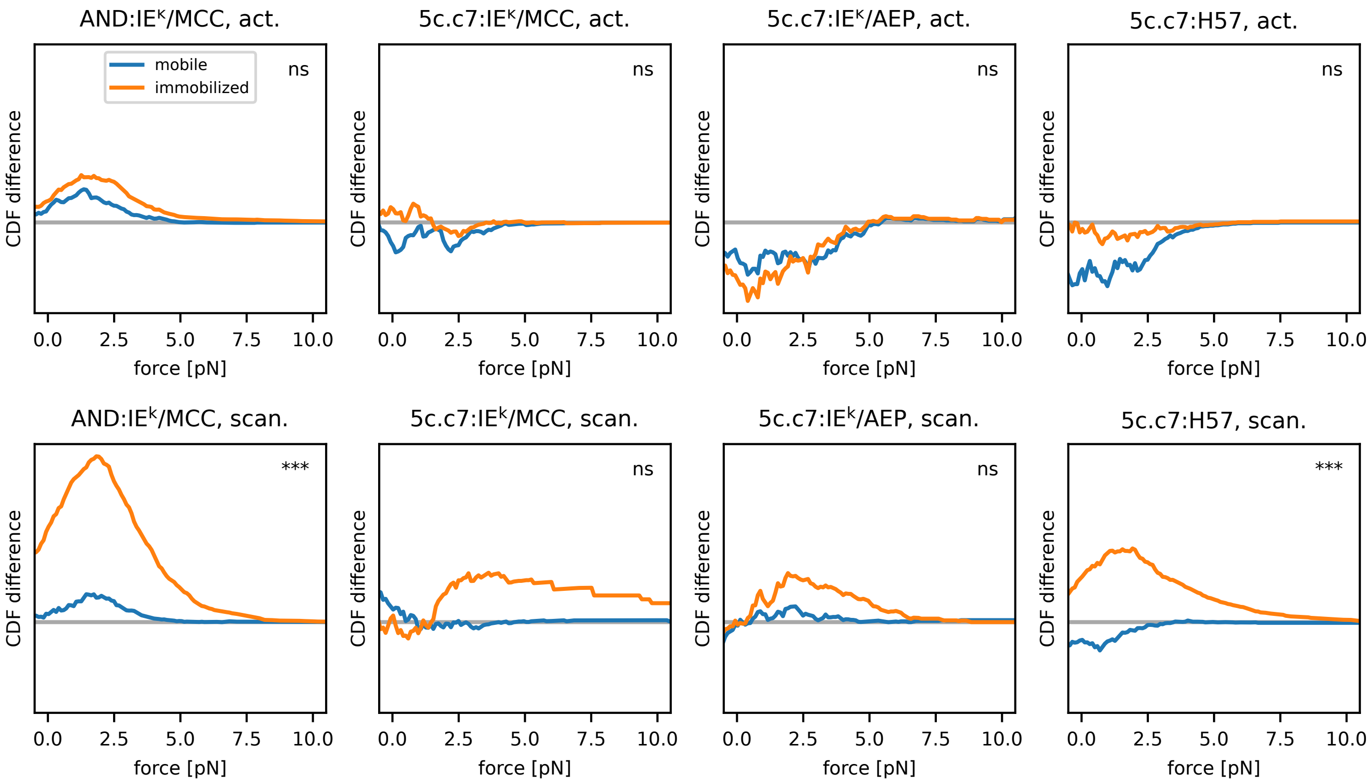

Supplementary figure 6: *Comparison of the cumulative density function (CDF) of the mobile and immobilized fraction* (no-cell background was subtracted). Significant differences to the no-cell data are indicated by the asterisks in the corner of each plot. $*$ – p-value < 0.05; $**$ – p-value < 0.01; $***$ – p-value < 0.001; ns – not significant. P-values were calculated employing one-sided KS tests adjusted for dependent datapoints within trajectories (see Methods for details). The number of recorded trajectories and mice are summarized in supplementary table 3. Source data are provided as a Source Data file.

2 Supplementary tables

		counts				high-force fraction			high-force quartiles [pN]		
	p-value	data points	tracks	videos	mice	threshold	proportion	error	Q1	Q2	Q3
5c.c7:H57, activating fluid-phase SLB	0.6183	911	49	44	2	N/A	N/A	N/A	N/A	N/A	N/A
5c.c7:H57, activating gel-phase SLB	0.0001	110569	3808	1584	18	0.65	0.11	0.01	2.6	4.0	5.7
5c.c7:H57, scanning fluid-phase SLB	0.0176	8550	320	226	4	0.64	0.06	0.02	3.0	4.2	5.9
5c.c7:H57, scanning gel-phase SLB	0.0001	23412	1029	543	7	0.63	0.11	0.01	4.2	5.5	7.2
5c.c7:IE^k/AEP, activating fluid-phase SLB	0.8094	2731	134	79	1	N/A	N/A	N/A	N/A	N/A	N/A
5c.c7:IE^k/AEP, activating gel-phase SLB	0.0009	21443	1091	339	3	0.65	0.06	0.01	2.0	3.0	4.9
5c.c7:IE^k/AEP, scanning fluid-phase SLB	0.3412	3054	143	111	2	N/A	N/A	N/A	N/A	N/A	N/A
5c.c7:IE^k/AEP, scanning gel-phase SLB	0.3128	26197	1116	334	3	N/A	N/A	N/A	N/A	N/A	N/A
5c.c7:IE^k/MCC, activating fluid-phase SLB	0.0562	1267	113	98	2	N/A	N/A	N/A	N/A	N/A	N/A
5c.c7:IE^k/MCC, activating gel-phase SLB	0.0001	79193	2628	1197	10	0.65	0.04	0.01	3.3	4.3	5.6
5c.c7:IE^k/MCC, scanning fluid-phase SLB	0.0037	864	75	66	2	0.62	0.02	0.02	0.9	2.2	3.9
5c.c7:IE^k/MCC, scanning gel-phase SLB	0.3883	34404	1054	553	6	N/A	N/A	N/A	N/A	N/A	N/A
AND:IE^k/MCC, activating fluid-phase SLB	0.0125	14972	482	289	4	0.62	0.03	0.01	1.8	2.8	4.0
AND:IE^k/MCC, activating gel-phase SLB	0.0001	58235	1714	529	5	0.65	0.14	0.01	3.9	5.0	6.5
AND:IE^k/MCC, scanning fluid-phase SLB	0.0002	11283	409	239	4	0.62	0.06	0.02	2.5	3.4	4.5
AND:IE^k/MCC, scanning gel-phase SLB	0.0001	8142	292	185	4	0.64	0.08	0.02	2.2	3.1	4.6

Supplementary table 1: Summary of statistics for all recorded molecular force data sets. P-values were calculated employing one-sided KS tests adjusted for dependent datapoints within trajectories (see Methods for details).

	time after seeding		counts				high-force fraction		
	start [min]	end [min]	data points	tracks	videos	mice	threshold	proportion	error
5c.c7:H57, activating fluid-phase SLB	0	10	567	30	28	2	N/A	N/A	N/A
5c.c7:H57, activating fluid-phase SLB	10	20	344	19	16	2	N/A	N/A	N/A
5c.c7:H57, activating gel-phase SLB	0	10	59442	1988	796	18	0.65	0.09	0.01
5c.c7:H57, activating gel-phase SLB	10	20	44948	1540	640	18	0.65	0.13	0.01
5c.c7:H57, scanning fluid-phase SLB	0	10	3590	151	102	4	0.64	0.08	0.03
5c.c7:H57, scanning fluid-phase SLB	10	20	3823	136	98	4	0.64	0.05	0.02
5c.c7:H57, scanning gel-phase SLB	0	10	7307	317	193	7	0.63	0.12	0.02
5c.c7:H57, scanning gel-phase SLB	10	20	7508	229	140	7	0.63	0.03	0.02
AND:IE^k/MCC, activating fluid-phase SLB	0	10	6247	220	130	4	0.62	0.04	0.01
AND:IE^k/MCC, activating fluid-phase SLB	10	20	8361	247	150	4	0.62	0.03	0.01
AND:IE^k/MCC, activating gel-phase SLB	0	10	24180	705	219	5	0.65	0.12	0.01
AND:IE^k/MCC, activating gel-phase SLB	10	20	28945	861	271	5	0.65	0.15	0.02
AND:IE^k/MCC, scanning fluid-phase SLB	0	10	5249	183	106	4	0.62	0.07	0.03
AND:IE^k/MCC, scanning fluid-phase SLB	10	20	4702	170	99	4	0.62	0.06	0.02
AND:IE^k/MCC, scanning gel-phase SLB	0	10	4329	150	94	4	0.64	0.11	0.03
AND:IE^k/MCC, scanning gel-phase SLB	10	20	3441	125	80	4	0.64	0.05	0.02

Supplementary table 2: Summary of statistics for recorded molecular force data sets split by time since cell seeding. P-values were calculated employing one-sided KS tests adjusted for dependent datapoints within trajectories (see Methods for details).

		counts				high-force fraction		
	p-value	data points	tracks	videos	mice	threshold	proportion	error
5c.c7:H57, activating, immobilized sensors	0.5452	705	29	25	2	0.63	0.00	0.02
5c.c7:H57, activating, mobile sensors	0.4864	175	11	11	2	0.63	−0.04	0.01
5c.c7:H57, scanning, immobilized sensors	0.001	5280	177	143	4	0.64	0.09	0.03
5c.c7:H57, scanning, mobile sensors	0.883	3156	106	92	4	0.64	−0.00	0.01
5c.c7:IE^{k}/AEP, activating, immobilized sensors	0.8446	945	38	31	1	0.45	−0.00	0.03
5c.c7:IE^{k}/AEP, activating, mobile sensors	0.7913	1712	73	55	1	0.45	−0.00	0.02
5c.c7:IE^{k}/AEP, scanning, immobilized sensors	0.2848	985	31	27	2	0.58	0.05	0.05
5c.c7:IE^{k}/AEP, scanning, mobile sensors	0.5468	2013	91	81	2	0.58	0.00	0.01
5c.c7:IE^{k}/MCC, activating, immobilized sensors	0.1517	696	36	36	2	0.62	−0.01	0.01
5c.c7:IE^{k}/MCC, activating, mobile sensors	0.2239	452	33	32	2	0.62	−0.02	0.01
5c.c7:IE^{k}/MCC, scanning, immobilized sensors	0.2956	191	13	12	2	0.62	0.06	0.07
5c.c7:IE^{k}/MCC, scanning, mobile sensors	0.0027	613	42	38	2	0.62	0.00	0.01
AND:IE^{k}/MCC, activating, immobilized sensors	0.0077	7895	226	183	4	0.62	0.05	0.01
AND:IE^{k}/MCC, activating, mobile sensors	0.0712	6993	229	156	4	0.62	0.02	0.01
AND:IE^{k}/MCC, scanning, immobilized sensors	0.0001	2976	76	66	4	0.62	0.18	0.05
AND:IE^{k}/MCC, scanning, mobile sensors	0.1629	8196	294	181	4	0.62	0.02	0.01

Supplementary table 3: Summary of statistics for recorded molecular force data sets split by sensor mobility. P-values were calculated employing one-sided KS tests adjusted for dependent datapoints within trajectories (see Methods for details).

	SLB	T [°C]	lifetime [s] estimate	lifetime [s] error	recording intervals [s]	counts by interval: frames per movie	counts by interval: recorded tracks
5c.c7:IE^k/AEP							
0	gel-phase	23	152.2	27.0	1.0, 2.5, 5.0, 7.5	180, 75, 40, 35	148, 162, 80, 161
0	fluid-phase	23	191.8	78.5	1.0, 2.5, 5.0, 7.5	180, 75, 40, 35	74, 105, 91, 75
1	gel-phase	23	102.2	15.2	1.0, 2.5, 5.0, 7.5	180, 75, 40, 35	157, 175, 219, 218
1	fluid-phase	23	88.9	12.1	1.0, 2.5, 5.0, 7.5	180, 75, 40, 35	151, 97, 185, 158
5c.c7:IE^k/MCC							
2	gel-phase	27	4.5	0.8	0.05, 0.1, 0.25, 0.5, 1.0	1000, 500, 200, 100, 50	32, 51, 49, 54, 50
2	fluid-phase	27	4.1	0.5	0.05, 0.1, 0.25, 0.5	1000, 500, 200, 100	160, 149, 125, 89
3	gel-phase	27	4.3	0.8	0.05, 0.175, 0.5, 1.5	500, 250, 100, 50	19, 24, 39, 43
3	fluid-phase	27	5.3	0.8	0.05, 0.175, 0.5, 1.5	500, 250, 100, 50	38, 42, 56, 52
4	gel-phase	27	4.2	1.0	0.05, 0.1, 0.25, 0.5, 1.0, 1.5	500, 250, 150, 75, 25, 16	12, 9, 21, 35, 15, 11
4	fluid-phase	27	4.3	0.4	0.05, 0.1, 0.25, 0.5, 1.0, 1.5	500, 250, 150, 75, 30, 20	117, 138, 133, 69, 90, 67
5	gel-phase	23	18.5	2.1	0.1, 0.25, 0.5, 1.0, 2.0	500, 200, 100, 50, 30	149, 254, 181, 188, 147
5	fluid-phase	23	13.5	1.0	0.1, 0.25, 0.5, 1.0, 2.0	500, 200, 100, 50, 30	256, 408, 254, 316, 320
6	gel-phase	23	11.6	1.5	0.1, 0.25, 0.5, 1.0, 2.0	500, 200, 100, 50, 30	149, 107, 103, 88, 89
6	fluid-phase	23	10.1	0.7	0.1, 0.25, 0.5, 1.0, 2.0	500, 200, 100, 50, 30	344, 327, 360, 327, 213
7	gel-phase	23	7.0	1.1	0.1, 0.25, 0.5, 1.0, 2.0	500, 200, 100, 50, 30	75, 46, 103, 37, 33
7	fluid-phase	23	13.6	1.3	0.1, 0.25, 0.5, 1.0, 2.0	500, 200, 100, 50, 30	191, 211, 203, 180, 188
AND:IE^k/MCC							
8	gel-phase	27	28.5	5.5	0.25, 1.0, 2.0, 4.0, 8.0	300, 75, 50, 25, 15	42, 43, 29, 3, 19
8	fluid-phase	27	27.1	6.6	0.25, 1.0, 2.0, 4.0, 8.0	300, 75, 50, 25, 15	25, 16, 11, 14, 6
9	gel-phase	27	19.0	3.7	0.25, 1.0, 2.0, 4.0	300, 75, 50, 25	25, 14, 19, 31
9	fluid-phase	27	21.9	5.2	0.25, 1.0, 2.0, 4.0, 8.0	300, 75, 50, 25, 15	21, 14, 15, 13, 8
10	gel-phase	27	29.0	3.1	0.25, 0.5, 1.0, 2.0, 4.0	400, 200, 100, 75, 35	140, 112, 94, 153, 82
10	fluid-phase	27	35.4	7.0	0.25, 1.0, 2.0, 3.0, 4.0	300, 75, 75, 50, 35	63, 40, 24, 17, 16
11	gel-phase	23	75.6	12.3	0.25, 0.5, 1.0, 2.5, 5.0	500, 250, 180, 75, 40	80, 97, 87, 114, 70
11	fluid-phase	23	89.7	24.4	0.25, 0.5, 1.0, 2.5, 5.0	500, 250, 180, 75, 40	59, 77, 43, 64, 28
12	gel-phase	23	88.3	15.6	0.25, 0.5, 1.0, 2.5, 5.0	500, 250, 180, 75, 40	155, 146, 142, 170, 143
12	fluid-phase	23	69.7	14.5	0.25, 0.5, 1.0, 2.5, 5.0	500, 250, 180, 75, 40	122, 180, 170, 150, 73

Supplementary table 4: Summary of statistics for all recorded lifetime data sets. For gel-phase and fluid-phase conditions in each experimental set (indicated by matching numbers in the first column), cells from the same mouse were used. For every receptor:ligand pair, each experimental set was recorded using cells from a different mouse.

	drug	T [°C]	lifetime [s] estimate	lifetime [s] error	recording intervals [s]	counts by interval: frames per movie	counts by interval: recorded tracks
0	Cyto-D	23	11.1	1.8	0.1, 0.25, 0.5, 1.0, 2.0	500, 200, 100, 50, 30	142, 22, 34, 13, 73
1	Cyto-D	23	12.5	2.0	0.1, 0.25, 0.5, 1.0, 2.0	500, 200, 100, 50, 30	104, 99, 53, 60, 72
1	DMSO	23	9.9	1.7	0.1, 0.25, 0.5, 1.0, 2.0	500, 200, 100, 50, 30	72, 136, 153, 100, 59

Supplementary table 5: Summary of statistics for recorded lifetime data sets upon actin cytoskeleton disruption. For gel-phase and fluid-phase conditions in each experimental set (indicated by matching numbers in the first column), cells from the same mouse were used. Each experimental set was recorded using cells from a different mouse.
