# Supplementary material for: CD4+T-cells create a stable mechanical environment for force-sensitive TCR:pMHC interactions
Source: Nat Commun. 2025 Aug 15;16:7577. doi: 10.1038/s41467-025-62104-2 (PMC12354883; doi:10.1038/s41467-025-62104-2)
Supplement: Supplementary file 2 — Reporting Summary [file 41467_2025_62104_MOESM2_ESM.pdf]

nature portfolio

Corresponding author(s): Göhring

Last updated by author(s): Jul 3, 2025

Reporting Summary

Nature Portfolio wishes to improve the reproducibility of the work that we publish. This form provides structure for consistency and transparency in reporting. For further information on Nature Portfolio policies, see our Editorial Policies and the Editorial Policy Checklist.

Statistics

For all statistical analyses, confirm that the following items are present in the figure legend, table legend, main text, or Methods section.

n/a	Confirmed	
☐	☒	The exact sample size (*n*) for each experimental group/condition, given as a discrete number and unit of measurement
☐	☒	A statement on whether measurements were taken from distinct samples or whether the same sample was measured repeatedly
☐	☒	The statistical test(s) used AND whether they are one- or two-sided<br>*Only common tests should be described solely by name; describe more complex techniques in the Methods section.*
☒	☐	A description of all covariates tested
☒	☐	A description of any assumptions or corrections, such as tests of normality and adjustment for multiple comparisons
☐	☒	A full description of the statistical parameters including central tendency (e.g. means) or other basic estimates (e.g. regression coefficient) AND variation (e.g. standard deviation) or associated estimates of uncertainty (e.g. confidence intervals)
☐	☒	For null hypothesis testing, the test statistic (e.g. *F*, *t*, *r*) with confidence intervals, effect sizes, degrees of freedom and *P* value noted<br>*Give P values as exact values whenever suitable.*
☒	☐	For Bayesian analysis, information on the choice of priors and Markov chain Monte Carlo settings
☒	☐	For hierarchical and complex designs, identification of the appropriate level for tests and full reporting of outcomes
☒	☐	Estimates of effect sizes (e.g. Cohen's *d*, Pearson's *r*), indicating how they were calculated

Our web collection on statistics for biologists contains articles on many of the points above.

Software and code

Policy information about availability of computer code

Data collection	SDT-control (custom-made microscope controller software), MetaMorph Software (version 7.10.1.161)
Data analysis	We provide the Python code for single-molecule FRET analysis v3.0 (https://github.com/schuetzgroup/fret-analysis, DOI: 10.5281/zenodo.4604567) as well as the underlying Python library (https://github.com/schuetzgroup/sdt-python, DOI: 10.5281/zenodo.4604495). Lifetime Software is available under https://github.com/schuetzgroup/smfret-bondtime, DOI: 10.5281/zenodo.12571064. In-house developed code implemented in Matlab R2019b was used to analyze calcium data (available on request).<br>Python Libraries: sdt-python (v19.0.2), smfret-bondtime (v1.0.1).<br>Code for figures: https://doi.org/10.48436/dccvp-w7q74

For manuscripts utilizing custom algorithms or software that are central to the research but not yet described in published literature, software must be made available to editors and reviewers. We strongly encourage code deposition in a community repository (e.g. GitHub). See the Nature Portfolio guidelines for submitting code & software for further information.

Data

Policy information about availability of data

All manuscripts must include a data availability statement. This statement should provide the following information, where applicable:

- Accession codes, unique identifiers, or web links for publicly available datasets
- A description of any restrictions on data availability
- For clinical datasets or third party data, please ensure that the statement adheres to our policy

The data generated in this study have been deposited in the TU Wien Research Data database under accession code dccvp-w7q74 (URL: https://doi.org/10.48436/dccvp-w7q74)

Research involving human participants, their data, or biological material

Policy information about studies with human participants or human data. See also policy information about sex, gender (identity/presentation), and sexual orientation and race, ethnicity and racism.

Reporting on sex and gender	*Use the terms sex (biological attribute) and gender (shaped by social and cultural circumstances) carefully in order to avoid confusing both terms. Indicate if findings apply to only one sex or gender; describe whether sex and gender were considered in study design; whether sex and/or gender was determined based on self-reporting or assigned and methods used. Provide in the source data disaggregated sex and gender data, where this information has been collected, and if consent has been obtained for sharing of individual-level data; provide overall numbers in this Reporting Summary. Please state if this information has not been collected. Report sex- and gender-based analyses where performed, justify reasons for lack of sex- and gender-based analysis.*
Reporting on race, ethnicity, or other socially relevant groupings	*Please specify the socially constructed or socially relevant categorization variable(s) used in your manuscript and explain why they were used. Please note that such variables should not be used as proxies for other socially constructed/relevant variables (for example, race or ethnicity should not be used as a proxy for socioeconomic status). Provide clear definitions of the relevant terms used, how they were provided (by the participants/respondents, the researchers, or third parties), and the method(s) used to classify people into the different categories (e.g. self-report, census or administrative data, social media data, etc.) Please provide details about how you controlled for confounding variables in your analyses.*
Population characteristics	*Describe the covariate-relevant population characteristics of the human research participants (e.g. age, genotypic information, past and current diagnosis and treatment categories). If you filled out the behavioural & social sciences study design questions and have nothing to add here, write "See above."*
Recruitment	*Describe how participants were recruited. Outline any potential self-selection bias or other biases that may be present and how these are likely to impact results.*
Ethics oversight	*Identify the organization(s) that approved the study protocol.*

Note that full information on the approval of the study protocol must also be provided in the manuscript.

Field-specific reporting

Please select the one below that is the best fit for your research. If you are not sure, read the appropriate sections before making your selection.

☒ Life sciences ☐ Behavioural & social sciences ☐ Ecological, evolutionary & environmental sciences

For a reference copy of the document with all sections, see nature.com/documents/nr-reporting-summary-flat.pdf

Life sciences study design

All studies must disclose on these points even when the disclosure is negative.

Sample size	Sample sizes were chosen in agreement with the observed variabilities in the samples and previous experience. No statistical method was used to predetermine sample size.
Data exclusions	Single-molecule FRET data was filtered as described in the method section of the manuscript. Exclusion criteria were established a priori.
Replication	Unless explicitly stated, all data shown were obtained from at least 2 biological independent experiments. All replications were included, if they were passing experimental controls for the biological functional state of the cells, as well as technical controls.
Randomization	Allocating samples into experimental groups was not applicable since primary T-cells from two mouse strain were used. No specific method for randomization was used.
Blinding	Allocating samples into experimental groups was not applicable. Blinding was therefore not relevant to this study.

Reporting for specific materials, systems and methods

We require information from authors about some types of materials, experimental systems and methods used in many studies. Here, indicate whether each material, system or method listed is relevant to your study. If you are not sure if a list item applies to your research, read the appropriate section before selecting a response.

Materials & experimental systems

n/a	Involved in the study
☐	☒ Antibodies
☒	☐ Eukaryotic cell lines
☒	☐ Palaeontology and archaeology
☐	☒ Animals and other organisms
☒	☐ Clinical data
☒	☐ Dual use research of concern
☒	☐ Plants

Methods

n/a	Involved in the study
☒	☐ ChIP-seq
☐	☒ Flow cytometry
☒	☐ MRI-based neuroimaging

Antibodies

Antibodies used	H57-scFv (refolded single chain antibody fragment derived from the TCRβ-reactive H57 monoclonal antibody, own production, refer to Huppa et al. Nature 2010, Brameshuber et al. Nature Immunology, 2018)
Validation	Validation data for H57-scFv is provided in the following manuscripts: Brameshuber et al. Nature Immunology, 2018 and Huppa et al., Nature, 2010. The modified antibody was tested for mobility after attachment to a supported lipid bilayer, and functional and specific binding was shown by a T-cell activation assay (ratiometric imaging of the calcium-sensitive dye Fura-2) in a similar fashion.

Animals and other research organisms

Policy information about studies involving animals; ARRIVE guidelines recommended for reporting animal research, and Sex and Gender in Research

Laboratory animals	Primary murine T cells were isolated from and AND TCR transgenic (Tg(TcrAND)53Hed, PMID: 2571940, B10.BR background) and 5c.c7 transgenic (Tg(Tcra5CC7,Tcrb5CC7)IWep, PMID: 1328464, B10.A background), male and female mice at age 12-16 weeks. Mouse house conditions: 22°C, 53% relative humidity, 12h dark-light cycle.
Wild animals	The study did not involve wild animals.
Reporting on sex	Experiments were conducted using animals of both genders in an even ratio.
Field-collected samples	The study did not involve samples collected from the field.
Ethics oversight	Mouse breeding and euthanasia were evaluated by the ethics committees of the Medical University of Vienna and approved by the Federal Ministry of Science, Research and Economy, BMWFW (BMWFW-66.009/0378-WF/V/3b/2016). All procedures to isolate lymphocytes and splenocytes from 8–12 weeks old gender-mixed mice were performed in accordance to Austrian law (Federal Ministry for Science and Research, Vienna, Austria), the guidelines of the Federation of Laboratory Animal Science Associations (FELASA), which match those of Animal Research Reporting In Vivo Experiments (ARRIVE), and the guidelines of the ethics committees of the Medical University of Vienna. Breeding and keeping of AND-TCR transgenic mice has been approved by the Government of Upper Bavaria, protocol 55.2-2532.Vet_02-21-4.

Note that full information on the approval of the study protocol must also be provided in the manuscript.

Plants

Seed stocks	*Report on the source of all seed stocks or other plant material used. If applicable, state the seed stock centre and catalogue number. If plant specimens were collected from the field, describe the collection location, date and sampling procedures.*
Novel plant genotypes	*Describe the methods by which all novel plant genotypes were produced. This includes those generated by transgenic approaches, gene editing, chemical/radiation-based mutagenesis and hybridization. For transgenic lines, describe the transformation method, the number of independent lines analyzed and the generation upon which experiments were performed. For gene-edited lines, describe the editor used, the endogenous sequence targeted for editing, the targeting guide RNA sequence (if applicable) and how the editor was applied.*
Authentication	*Describe any authentication procedures for each seed stock used or novel genotype generated. Describe any experiments used to assess the effect of a mutation and, where applicable, how potential secondary effects (e.g. second site T-DNA insertions, mosiacism, off-target gene editing) were examined.*

Flow Cytometry

Plots

Confirm that:

☒ The axis labels state the marker and fluorochrome used (e.g. CD4-FITC).

☒ The axis scales are clearly visible. Include numbers along axes only for bottom left plot of group (a 'group' is an analysis of identical markers).

☐ All plots are contour plots with outliers or pseudocolor plots.

☐ A numerical value for number of cells or percentage (with statistics) is provided.

Methodology

Sample preparation	For cell surface labelling, 5x105 T-cells were labelled with AF555-H57-scFV (250 µg/mL) for 15 min on ice, and washed 2 times in FACS buffer (1x PBS, 1% BSA, 0.02% NaN3). For titration experiments, the following mass of the AF555-H57-scFV was used: 20, 200, 600, 1800, 5400, and 16200 ng per 1 Mio cells. For time curve experiments 1800 ng per 1 Mio T-cells was used. After washing T-cells were placed on ice, room temperature or into a 37°C water bath. Samples were removed from the respective temperatures after the following intervals (0, 5, 10, 30, 60, and 120 min) and immediately analysed. Unlabelled cells were used as a reference and measured as separate sample.
Instrument	Samples were analyzed on the Cytek Aurora (Cytek Biosciences).
Software	Data derived from flow cytometry measurements were analyzed with the FlowJo v10 software (BD Biosciences).
Cell population abundance	We did not sort cells in this project.
Gating strategy	The lymphocytes were gated using FSC vs SSC. The singlets were gated based on the area vs height of FSC.

☐ Tick this box to confirm that a figure exemplifying the gating strategy is provided in the Supplementary Information.
